# Small Language Models for Early Detection of ADRD: Open-Source Language Models in Clinical Speech Classification

**DOI:** 10.1093/geroni/igaf122.4317

**Published:** 2025-12-31

**Authors:** Venkatanand ram Addepalli, Praveen Rao, Erich Kummerfeld, Andrew Kiselica, Knoo Lee

**Affiliations:** Institute of Data Science and informatics, University of Missouri, Columbia, Missouri, United States; University of Missouri, Columbia, Missouri, United States; University of Minnesota - Twin Cities, Twin Cities, Minnesota, United States; University of Georgia, Athens, Georgia, United States; University of Missouri - Columbia, Columbia, Missouri, United States

## Abstract

Large language models (LLMs) have shown promise in biomedical and clinical applications, including aging research, patient care, and translational geriatrics. However, their use is limited by costs, privacy risks, and HIPAA concerns. These challenges point to an alternative: Small Language Models (SLM; < 2 billion parameters) that run entirely on local hardware, reducing latency and dependence on external services. Recent open-source releases (e.g., DeepSeek R1) highlight the growing capability and efficiency of open-source models. These advances draw attention to SLMs, which are inherently efficient and easier to deploy in local clinical settings. This underscores the need to evaluate smaller, open-source language models on similar tasks. In this study, we evaluated four SLMs (DeepSeek Coder, GPT-2, Microsoft Phi-3, SmolLM2) on 398 transcripts from the Pitt Corpus, comprising dementia patients and control participants evaluated with the Cookie Theft picture description task. To our knowledge, this is the first on-premise deployment of an SLM directly on linguistic features of transcripts on those with ADRD, demonstrating comparable dementia classification accuracy to LLMs under HIPAA-compliant, low-latency conditions. Models were trained and tested on a subset of the Pitt corpus. The best-performing model (GPT-2) reached 78.3% accuracy with inference times under three seconds. This balance of accuracy with efficiency demonstrates the feasibility of locally-hosted SLMs as privacy-preserving tools for dementia detection. Our findings suggest that SLMs may offer practical pathways for deploying AI in aging research and clinical care. Future work will assess real-time speech analysis with integration of multimodal data for comprehensive geriatric assessment.

